# Failure modes and downtime of radiotherapy LINACs and multileaf collimators in Indonesia

**DOI:** 10.1002/acm2.13756

**Published:** 2022-08-24

**Authors:** Gregory Sadharanu Peiris, Supriyanto Ardjo Pawiro, Muhammad Firmansyah Kasim, Suzie Lyn Sheehy

**Affiliations:** ^1^ School of Physics University of Melbourne Melbourne Victoria Australia; ^2^ Department of Physics, Faculty of Mathematics and Natural Sciences Universitas Indonesia Depok City Indonesia; ^3^ Department of Physics University of Oxford Oxford England

**Keywords:** downtime, faults, LINAC, multi‐leaf collimator, radiotherapy

## Abstract

**Background and purpose:**

The lack of equitable access to radiotherapy (RA) linear accelerators (LINACs) is a substantial barrier to cancer care in low‐ and middle‐income countries (LMICs). These nations are expected to bear up to 75% of cancer‐related deaths globally by 2030. State‐of‐the‐art LINACs in LMICs experience major issues in terms of robustness, with mechanical and electrical breakdowns resulting in downtimes ranging from days to months. While existing research has identified the higher failure frequency and downtimes between LMICs (Nigeria, Botswana) compared to high‐income countries (HICs, the UK), there has been a need for additional data and study particularly relating to multileaf collimators (MLCs).

**Materials and methods:**

This study presents for the first time the analysis of data gathered through a dedicated survey and workshop including participants from 14 Indonesian hospitals, representing a total of 19 LINACs. We show the pathways to failure of radiotherapy LINACs and frequency of breakdowns with a focus on the MLC subsystem.

**Results:**

This dataset shows that LINACs throughout Indonesia are out of operation for seven times longer than HICs, and the mean time between failures of a LINAC in Indonesia is 341.58 h or about 14 days. Furthermore, of the LINACs with an MLC fitted, $59.02_{-1.61}^{+1.98}$% of all mechanical faults are due to the MLC, and $57.14_{-1.27}^{+0.78}$ % of cases requiring a replacement component are related to the MLC.

**Conclusion:**

These results highlight the pressing need to improve robustness of RT technology for use in LMICs, highlighting the MLC as a particularly problematic component. This work motivates a reassessment of the current generation of RT LINACs and demonstrates the need for dedicated efforts toward a future where cancer treatment technology is robust for use in all environments where it is needed.

## 1 INTRODUCTION

Radiation therapy is an effective and ubiquitous form of treatment used in modern cancer care.^1^, ^2^ Where radiotherapy is available, it is used in 40% of all successfully treated cancer cases.^3^ State‐of‐the‐art radiotherapy relies on using a compact linear accelerator—or LINAC—with a typical operating lifetime of around 10–12 years in high‐income countries (HICs) clinics. There is a dramatic shortfall of LINACs in low‐ and middle‐income countries (LMICs): it has been estimated that that around 90% of patients in low income countries do not have access to radiotherapy, and that at least 5000 new LINACs in the next two or three decades are needed to meet the increasing burden of cancer in these regions.^4^, ^5^, ^6^, ^7^ Despite new facilities being installed, the shortfall is getting worse as there is an ever increasing proportion of cancer‐related deaths attributed to LMICs, expected to be as high as 75% of all global cases by 2030.^1^, ^2^ At least 5000 new LINACs are needed by 2035 to meet the increasing burden of cancer in these regions, yet if one well‐staffed LINAC is installed per week, this will take 100 years.

Solving this global healthcare challenge requires international collaboration and partnership far beyond existing approaches: not just for LINAC technology, but also to address staffing needs and build resilient healthcare systems. The many issues are discussed in detail in the literature.^8^ In recent years, a concerted effort to build such a trusted global collaboration has been established by the International Cancer Expert Corps (ICEC),^9^ using a partnership approach including promoting greater recognition for individuals and institutions, producing peer‐reviewed scientific, clinical and policy journals and most crucially the mentorship and support of early‐career experts. ICEC in collaborative effort with CERN and the UK's Science and Technology Facilities Council convened a series of workshops starting in 2015^10^ with a wide range of experts from accelerator technology to global cancer policy, with many attendees from LMICs. This has helped grow a trusted global network of collaborators^11^ including all 28 African nations offering LINAC‐based radiotherapy. The present study is motivated by the findings in these workshops and the studies, which have emerged.

From a technology perspective, it is now clear that current LINAC technology is not well‐suited to meet the shortfall in LMICs, with studies into the quality of cancer care in sub‐Saharan Africa showing LINACs “often do not function well in the adverse conditions encountered in LMICs.”^5^ This observation has been substantiated in further research, which found that LINAC breakdowns in LMICs are more frequent and on average much longer than in HICs, with downtimes ranging from days to months.^12^, ^13^

**FIGURE 1:**
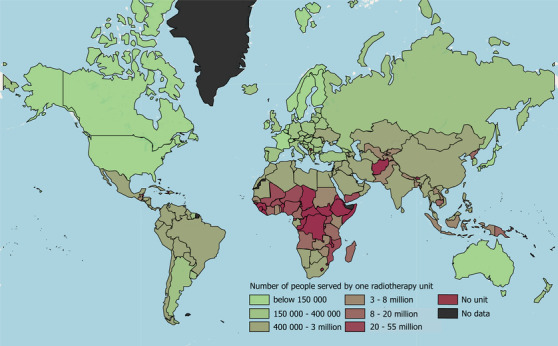
Many low‐ and middle‐income countries (LMICs) have limited access to radiotherapy units with some countries only having one unit to serve over 20 million people, displayed here.

Regions of low GDP are particularly vulnerable to LINAC breakdowns, as they do not have sufficient facilities to handle their patient load, as seen in Figure 1. A LINAC fault in a well‐resourced HIC hospital may result in a patient being treated on a different machine or a slight scheduling change, but in an LMIC hospital with few LINACs—sometimes just one machine—breakdowns can be far more disruptive. Patients may either miss out on treatment or have to travel large distances to access the next available facility, which may well be in another country.^14^ In addition to the issue of robustness, the upfront cost and maintenance costs of LINACs can be a substantial impediment in LMICs when considering the relative expense compared to, for example, personnel costs.

In considering possible solutions for radiotherapy in LMICs, one suggestion would be to use cobalt‐60 or caesium‐137 radiation sources.^5^, ^15^ Although these are inherently reliable and have fewer moving parts, due to safety and nuclear security issues they are not considered to be a suitable technology option compared to LINACs for LMICs and are thus not considered in this study. For equitable access, technological solutions for LMICs need to be world standard and deliver the same quality of care as HICs. As highlighted by Coleman et al., this also encourages experts to work and remain in LMICs.^5^

Many LINAC faults appear to be linked to inconsistent power supply and insufficient preventative maintenance, along with inaccessible spare parts and inadequate access to expert training courses.^12^ One of the particularly troublesome components is the multileaf collimator (MLC), which is also reported to break down frequently in high income countries.^13^, ^16^ However, to date research has not sufficiently investigated the extent to which MLCs are problematic in the delivery of radiotherapy in LMICs, primarily due to the lack of machines fitted with MLCs in the previous study based in sub‐Saharan Africa.^13^

In this work, we analyze the recorded breakdown data of 19 LINACs from 14 hospitals across Indonesia, focusing on the MLC. Where existing attempts to address the issues associated with RT in LMICs have relied on anecdotal failure data or estimates of downtime based on recollections,^10^ this work, for the first time, takes a data‐driven approach. Using a novel dataset and survey responses provided by facilities across Indonesia, we look directly at the timing, nature, and possible cause of LINAC breakdowns. The advantage of having all the data from a single country is the control of variables such as GDP and climate; all breakdowns across the various hospitals can be used as a representative sample of Indonesia as a whole. This study is therefore one of the first to use a data driven approach to analyse the impact of the MLC downtime in LMICs and to compare this to existing HIC data.

## 2 MATERIAL AND METHODS

### 2.1 Collection of Indonesian hospital data

The data used in this study were collected during a specialized workshop held in Jakarta, Indonesia in July 2019^1^. Representatives (mostly medical physicists) from 14 hospitals across Indonesia were invited to use their LINAC logbook data to populate a template spreadsheet provided by the authors with instructions in both English and Indonesian. A survey was also conducted to collect relevant contextual data including staffing levels, number of LINACs and typical operating hours, and issues around power, humidity, and external environmental issues. The facilities involved in the study and the number of RT LINACs at each are presented in Table 1 with some data spanning as far back as 2000. Each facility provided one or more logbooks of the machine's failures and maintenance as recorded by medical physicists or engineers.

**TABLE 1:** Summary of all Indonesian hospitals used in the research and their number of LINACs

**Label**	**Hospital**	**Number of LINACs**	**Years of data collected**	**Number of patients**
A	Pasar Minggu Hospital	1	2017–2019	10 000
B	Pusat Pertamina Central Hospital	1	2006–2019	45 300
C	Nusa Tenggara Barat Province Hospital	1	2018–2019	3400
D	Persahabatan Hospital	1	2017–2019	16 800
E	Dr Cipto Mangunkusumo General Hospital	5	2000–2019	182 400
F	Dr Hasan Sadikin Central General Hospital	1	2004–2018	208 800
G	Abdul Wahab Sjahranie Hospital	1	2016–2019	49 400
H	Kanker Gading Pluit Hospital	1	2013–2019	11520
J	Adam Malik General Hospital	1	2013–2018	85 800
K	Andalas University Hospital	1	2019	4500
L	Murni Teguh Memorial Hospital	2	2015–2019	58 800
M	Vina Estetica Hospital	1	2014–2019	53 400
N	MRCCC Siloam Hospital	2	2014–2019	65 000
P	Indriati Hospital	1	2017–2019	5700

*Note*: Number of Patients is an estimate extrapolated per facility across the years of data provided.

The date and duration of each fault was recorded as “downtime,” and the duration between faults was recorded as “time between failures.” Each downtime duration is sorted into one of three categories:

1. A ≤5 min,
2. 5 mins < B ≤ 59 min,
3. C > 59min.

A LINAC's “uptime” or “runtime” is defined by the total hours of operation from commissioning to decommissioning.

This categorization has been used in previous downtime studies and is justified based on disruption to workflow and irradiation capability.^13^, ^17^ Faults in the LINACs were further categorized by their cause for failure: (mechanical, electrical, board, cabling, external, parameter drift). Mechanical faults include anything motion related; electrical faults are related to electrical issues like blown fuses or power spikes; faults categorized as board faults relate to power boards; cabling faults relate to replacing or repairing damaged cables; faults in components such as the chiller, treatment couch, or other external elements are classed as external; and, any repairs related to recalibrating parameters, such as underdose rate interlocks are classified as parameter drift. The faults were also categorized by how the fault was corrected: (reset, replace, repair, calibrate). Reset includes reinitialising or restarting the machine; replace was used any time a component needed to be entirely or partially replaced; repair was when a broken component was either readjusted or fixed; and, calibrate was for remedies that required a recalibrating of parameters or positions.

The logbook data are used in conjunction with the survey data. This allows the failure data to be normalized accounting for the differences in the ranges of data. Logbook data did not include exact monitor unit data, so machine usage and “wear and tear” has to be estimated, and this typically occurs during patient treatment rather than quality assurance (QA) or start‐up tasks, so standardizing the data by patients treated is used. As such all relevant data have been normalized by 1000 patients treated. This contrasts with previous studies, which normalize by 1000 h of uptime as they did not have access to the average daily patient statistics. Smaller hospitals also treat fewer patients per day than those in more central areas hence normalizing by total number of patients is a fairer weighting. In our dataset, facilities, which have multiple LINACs, provided data on the average number of patients treated per machine each day (or more accurately patient fractions delivered).

The logbooks varied significantly in the details provided. There was variety in vendors and models of LINAC, and not all logbooks began at the commissioning of the machine. Most provided the date and time of faults, a brief description of the fault, how it was repaired and a duration of the downtime. The differences in record keeping habits between facilities made the automation of data collection and analysis challenging. As such all provided data were manually checked for quality, categorized, and entered into a csv formatted spreadsheet for processing with Python.

Within the logbook and survey data, information about the QA and preventative maintenance inspections (PMI) are present although similar to the breakdown data, inconsistent. Of the facilities that did provide their QA procedures, most were either an hour in the morning or several hours (between 2 and 5) on a weekend, but exact details were not recorded. The benefits of PMI are well documented for HICs^18^, ^19^; however there is not enough data to draw any meaningful conclusions about the effect of QA and PMI on LINAC breakdowns in LMICs. As such, we treat the QA procedures as consistent across facilities and study the breakdowns occurring in spite of operators best efforts in these measures.

This particular set of data gives us a deeper insight into the behaviour of RT machines in LMICs with information such as the type of fault and the method used to rectify the error. To analyze the large dataset (i.e., 4900 faults recorded across 19 Indonesian LINACs over 19 years), two methods paralleling Wroe's comparative study^13^ were implemented. The first categorizes and compares downtimes of LINACs and the time between failures and the second categorises the failure modes and methods used to repair LINACs.

### 2.2 Multileaf collimator subsystem data

As this study is focused on the MLC, instances of MLC‐related failures were isolated in order to investigate how frequently this subsystem failed and to analyze its overall impact on LINAC downtime. The downtime lengths, reason for failure, and resolution method are sorted using the same categories as described in Section II.I. Publicly available data pertaining to each type of LINAC were used to identify the number of MLC leaves and leaf width for each machine, recorded in Table 2.

**TABLE 2:** Types of LINACs commissioned at each facility and associated multileaf collimators (MLCs) parameters

**Label**	**Vendor and model**	**Year**	**Leaves**	**Leaf width (mm)**	**Downtime**	**MLC downtime**
A	Varian Trilogy	2017	120	5 + 10	23%	1.2%
B	Siemens Primus**^†^**	2006			11%	
C	Varian Clinac CX	2017	80	5 + 10	0.68%	13.4%
D	Elektra Precise	2016	80	10	4.9%	28.4%
E1	Varian Clinac 2100C*	2000	80	5 + 10	24%	5.7%
E2	Varian Clinac 2100C*	2001	80	5 + 10	30%	9.8%
E3	Elekta Synergy S	2006	80	10	17%	12.2%
E4	Elekta Synergy Platform	2008	80	10	13%	83.9%
E5	Varian Unique	2017	120	5	4.5%	49.8%
F	Elekta Precise**^†^**	2004				
G	Elekta Precise	–	80	10	6.4%	19.5%
H	Elekta Synergy Platform	2012	80	10	6.5%	22.0%
J	Elekta Precise	–	80	10	14%	26.8%
K	Varian Clinac CX	2016	80	5 + 10	9.0%	83.9%
L1	Elekta Synergy Platform	2013	80	10	0.95%	0.41%
L2	Elekta Versa HD	2017	160	5	19%	0.02%
M	Siemens Primus M	2011	58	10 + 65	14%	1.8%
N	Varian Clinac iX	2010	80	5 + 10	2.6%	27.6%
P	Varian Clinac iX	2017	80	5 + 10	1.5%	38.6%

*Note*: All MLCs have a field size of 40cm × 40cm. Leaf widths with two numbers denote inner and outer leaf width respectively.

*Machines that have been decommissioned at the hospital.

^†^Machines without an MLC. Hospital F's downtimes were unable to be calculated since no rectified time was provided, only incident time.

## 3 RESULTS

### 3.1 Analysis of downtime in Indonesia

The overview of LINACs in the study including percentage downtime caused by the MLC, number of MLC leaves, and leaf width is shown in Table 2. In Table 3, the LINAC faults include two machines (B, F), which do not have an MLC fitted. Although type C faults only make up 43% of all faults by number, they contribute to over 98% of the total downtime of all LINACs. While this implies that type C faults are more severe, it is caused in part by a lack of data on shorter interruptions as LMIC centres do not always record instances of type A and B faults. Were the Type A and B faults included, the impact on the data might be assumed to be negligible, given the orders of magnitude difference in total downtime. Type C faults contribute the most to downtime. While this seem trivial, when compared to an HIC, it is clear that A and B faults should contribute a higher proportion.

**TABLE 3:** Comparison of mean and median failure downtimes by downtime category in all LINACS and in the multileaf collimators (MLCs)

	**Category**	**Faults**	**Total downtime (hours)**	**Mean downtime (minutes)**	**Median downtime (minutes)**
LINAC	A	102	1.51 (0.002%)	1.65 ± 0.16	1
	B	2088	798.61 (1.19%)	22.95 ± 0.25	20
	C	1657	66052.43 (98.80%)	2391.76 ± 522.70	165
MLC	A	37	0.900 (0.01%)	1.46 ± 0.27	1
	B	733	297.43 (3.39%)	24.35 ± 0.42	23
	C	438	8463.20 (96.60%)	1159.34 ± 338.19	120

*Note*: A constitutes a downtime less than 5 min, B a downtime between 5 min and 1 h, and C downtimes are longer than an hour.

From the survey across all hospitals, the average daily LINAC operating time lasts 9.36 h in which an average of 37.6 patients are treated. Using this, the average downtime experienced by a LINAC from a type C fault is 4.2 ± 0.93 operating days. When only considering machines with an MLC fitted, 27.3% of all Type C faults are related to the MLC. For the MLC subsystem, the average downtime is shorter, at 2.06 ± 0.60 operating days.

Wroe's investigation of LINACs in the UK found that the mean downtime for a Type C fault is 338.8 min. In stark comparison, LINACs in Indonesia have a mean downtime of 2391.76 min: seven times longer than in the UK.

### 3.2 Overview of failure rates and reasons for failure

LINAC performance is quantified by the downtime of all the machines and the mean time between failure (MTBF). MTBF is a parameter regularly used in engineering reliability analysis to quantify and study faults.^20^, ^21^ The MTBF measures the average time between the resolution of one fault and the reporting of a subsequent one. In most mechanical systems, from installation to decommissioning one expects to see high failure rates early on and later on in a machine's lifetime and a lower constant failure rate through the middle, in a distribution of failures over time known as a “Bathtub Curve.” The MTBF usually considers only a machine's “useful life,”^20^, ^22^ since early failures and wear out due to fatigue can affect the mean. We note that in the breakdown data provided, there is no evidence of a clear bathtub curve to demarcate between regions of the machine's lifetime; thus all the data are used in the analysis.

**FIGURE 2:**
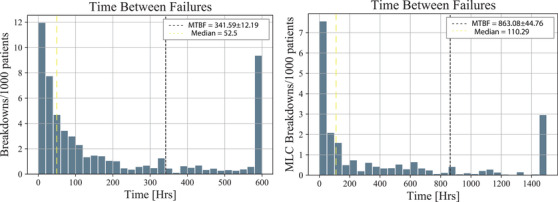
Histogram showing the time between failures for all LINACs (left) and just the multileaf collimators (MLCs) component (right). The mean time between failures (MTBF) is indicated by the black dotted line, and the median is given by the yellow dotted line. The overflow bin is 600 for the left and 1500 for the right.

From the data, the MTBF for a LINAC in Indonesia is 341.58 h, or about 36 operating days as seen in Figure 2. For an MLC in Indonesia, the MTBF is 863.08 h, or 92 operating days. More importantly, this means, on average, one fault happens for every 1369 patients that are treated on a LINAC and one fault in the MLC for every 3460 patients that are treated.

Another way to understand these data is to look at what fraction of the time the LINAC is unavailable due to failures: the total downtime divided by its total uptime, which can be seen in Table 2 ranging from just 0.68% to 30%. This range agrees with the weighted average for LINAC downtimes in Nigeria, which is 26.18%.^13^ Similarly, the contributions to the total downtime due to MLC‐related faults as a proportion of total LINAC downtime can also be found in Table 2, ranging from 0.02% to 83.9%. Different facilities thus have extremely varied experiences.

**FIGURE 3:**
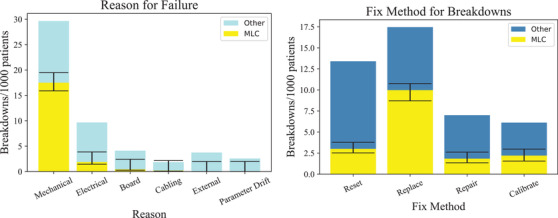
Histogram showing the most common reasons for failures (left) and the most common methods for fixing failures (right) across all LINACs in the Indonesian dataset normalised to the number of patients treated. The error bars are generated from the uncategorised data points.

Next, we look at the cause of faults of LINAC as a whole and the MLC. As shown in Figure 3, mechanical faults are the most common type of faults with 29.66 mechanical faults occurring for every 1000 patients treated in Indonesia. Of these, $59.02_{-1.61}^{+1.98}$% is related to the MLC subsystem.

Within the data, some faults and resolutions were left unfilled or lacked enough description to be categorized. We have used these data points as representative of errors in both recording and categorizing faults, in order to produce the error bars in Figure 3, described in Appendix A.

The most common method of fixing a faulty LINAC is by replacing a component: this occurs 17.46 times for every 1000 patients treated, in other words, only an average of 57 patients are treated before a component needs to be replaced. $57.14_{-1.27}^{+0.78}$ % of the replacements is due to the MLC. The most common components being replaced in the MLC are leaves, leaf motors, and T‐nuts, all of which are mechanical parts of the system. The majority of replaced parts outside the MLC are fuses and cables.

**FIGURE 4:**
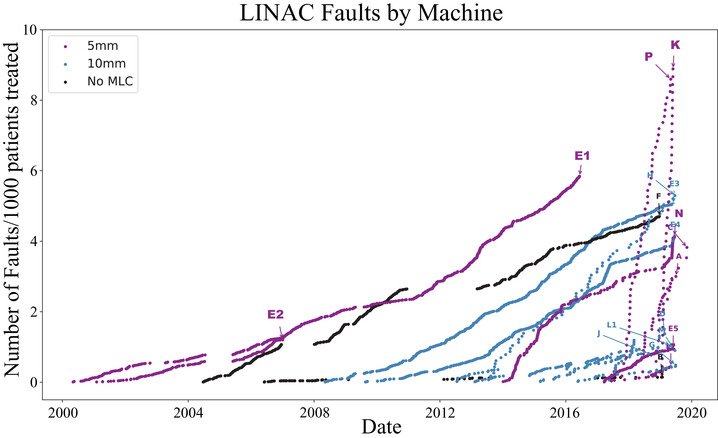
All faults displayed as a function of date per each hospital and grouped by leaf width in the multileaf collimators (MLCs). Labels correspond to the RT machines found in Table 2.

Figure 4 shows the cumulative faults where colour coding corresponds to the width of the innermost leaves in the MLC. A discussion of leaf width with respect to LINAC faults appears in detail later, in Section IV.I. To compare the data appropriately, the number of faults has been normalized by the number of patients treated at each facility. Note that hospitals, which do not treat a large number of patients, naturally become outliers when the normalization of “faults per 1000 patients” is used. This is clear in the two hospitals with near vertical lines (Hospital **K** and **P**). Hospital **C** also has a steep gradient due to the small number of patients, although it is harder to see in the figure.

## 4 DISCUSSION

The data used in this analysis rely on accurate log keeping and consistent entries of LINAC faults. All the conclusions drawn from the data have relied on thorough interactions with the hospital staff at a dedicated workshop, but in most cases this level of data accuracy is not guaranteed, which is one reason why such a study has not previously been produced. Even so, data entries varied significantly in the level of detail provided and often had years worth of data missing as evidenced by the large gaps in Figure 4. Some of these align with natural disasters, while others are seemingly random but are often related to staffing changes or staff workload. In Table 2, for instance, Hospital **F**’s downtime was not possible to calculate since no rectification time was provided. Standardizing or automating fault logging would improve future studies.

As mentioned earlier, the faults over time shown in Figure 4 do not show the expected shape for a cumulative bathtub curve. This suggests either that machines are being used longer than their suggested lifetime^23^ or that the failure rates in LMICs do not ease up over time. The reality may be that both factors are at play. It is well known in HIC's that due to advances in technology and software, LINACs get updated and replaced sooner than in LMICs.

With improved or automated record‐keeping, future studies are needed to investigate the mean times between specific failures. The present data hint that a build‐up of minor faults (Type **A** and **B** or Reset and Calibrate) occur before a disabling fault (Type **C** or Replace). To conclusively state this, however, would require a more rigorous record of minor faults. The issue of comprehensive record‐keeping and its value in improving hospital‐led interventions for LINAC operation and preventative maintenance was raised on multiple occasions by the Indonesian participants in the workshop.

A majority of downtime, especially for replacements, is spent waiting either for spare parts or for vendor engineers. This is experienced throughout other LMICs, particularly in African nations, where access to suitably trained personnel to maintain and repair of LINACs is harder than in HICs.^12^ In the survey, medical physicists at the various hospitals indicated that better training, better availability of vendor engineers, and easier access to spare parts would help improve uptime.

From Figure 2, the MTBF for LINACs through Indonesia was calculated to be 341.59 ± 12.19 h. However, the data are a heavily skewed distribution, and as such a better measure of the centre is the median, which in this case is 52.5 h or 5.61 operating days between failures.

The evidence from the Indonesian fault data suggests that LINACs have higher downtimes and failure rates in LMICs than in HICs. Although each LMIC is a unique environment, patterns are emerging among some of the common challenges, not least of all staffing and adequate training, and access to spare parts. Having a clear understanding of the similarities and differences between LMIC experiences can help address the global need for RT, but this is difficult when logbook records are not consistent anywhere in the world. Future attempts to combat the dramatic shortfall of LINACs will require data‐driven approaches from global collaborations to build and maintain a standardized knowledge base of how RT LINACs function and faulter in LMICs. Only then can we resolve this global challenge.

### 4.1 The multileaf collimator

The MLC contributes to a large portion of failures of LINACs in Indonesia. Of the machines which have an MLC, 25% ± 6.37% of the total downtime and 27.3% of the faults by number are due to the MLC. MLC faults in HICs are comparatively lower and contribute only 17.17% by downtime and 20.5% by number of faults.^13^ The downtime contribution is lower than the fault number contribution because MLCs often take less time to repair than other faults. A majority of the MLC failures are mechanical in nature due to the many moving parts, including at least 58 leaves, each with its own motor. To this effect, we can look at the contribution to downtime by the MLC from Table 2 as a function of leaf width. Omitting the outliers (83.9% from **E4** and **K**), 5 mm leaf widths account for 18.27 ± 6.5% (*N* = 8) of LINAC faults, while 10 mm leaves contribute 15.87% ± 4.3% (*N* = 7). Even though the average of the 10 mm is lower, the statistical sample is too low at present to conclude that leaf width (and thus number of leaves) is the key problem.

A direct comparison of machines with and without MLCs to quantify differences in failure rates and downtimes is not possible in this dataset since only two machines do not have an MLC; one of which, Hospital F, has provided no downtimes. Instead, a comparison between Wroe's findings and these results can be investigated. The two datasets can be compared only in terms of downtime, as Wroe's LMIC fault data are normalized per 1000 h uptime, as opposed per 1000 patients. The mean downtime for MLCs for *only* category **C** faults in the UK is 271.3 min compared to the 1159.34 ± 338.19 min for *all* category types in Indonesia. This corresponds to an MLC downtime 4.3 times longer in Indonesia compared to the HIC. This disparity would be even larger if the mean of all faults from the UK are used; thus the 4.3 is a lower bound. Another point of difference is that in the UK, type **B** and **C** MLC faults share around 45% of the total downtime, whereas in Indonesia, it is dominated by type **C** faults at 96.6%. Although this is in part due to longer wait times for spare parts and extended repair times, the high percentage is also due to a lack of type **A** and **B** records.

### 4.2 MLC alternatives

With the problems in the MLC persisting through all regions of the world,^6^, ^7^, ^12^, ^13^, ^24^ it is important to look at the possible alternatives for X‐ray collimation in future radiotherapy systems. Prior to the introduction of MLCs, LINACs used alloy block field shaping; however reverting to this method means an increase in treatment time and a reduction in prescribed dose being delivered.^25^, ^26^ An emerging alternative for X‐ray collimation is the scanning pencil‐beam high‐speed intensity modulated X‐ray source (SPHINX) collimator, which uses a 10 cm tungsten block with tumor size dependent tapered and diverging channels.^27^, ^28^ This removes the need for an MLC by driving the electrons to the most appropriate point on a bremsstrahlung target and having the diverging channels deliver the X‐rays to the tumor: effectively shaping the radiation to the tumor further upstream. The applicability of this technology should be studied in terms of its use in more challenging maintenance environments in LMICs.

An alternative that might cause the least amount of disruption to workflow is changing the parameters of the leaves or the design of the existing MLCs. By turning to reliability engineering, we can see that as the number of independent elements increase in a complex machine, the reliability decreases.^29^, ^30^ Specifically, “it is the active interfaces (i.e., moving surfaces) that are the most common regions for failures to occur.”^30^ Figure 3 shows mechanical faults as the prevailing issue, and it is established that leaf motors needing to be replaced accounts for a majority of the replacement resolutions. Having fewer leaves would decrease the number of leaf motors used and decrease the total contact surface area between leaves in an MLC. While this will ideally improve the machine's robustness, how it affects the quality of cancer treatment is a question for future studies.^31^, ^32^, ^33^

## 5 CONCLUSION

This study investigated the state of radiotherapy LINACs in Indonesia with a focus on the MLC subsystem and shows $59.02_{-1.61}^{+1.98}$% of mechanical faults and $57.14_{-1.27}^{+0.78}$ % of replacements are due to the MLC. A LINAC in Indonesia is down for seven times longer than one in the UK, and an MLC is down for 4.2 times longer. Although the MTBF of a LINAC is 341.58 h, the data are positively skewed, and thus the median is 52.5 h. This work supports and quantifies earlier research findings that LMICs are especially susceptible to mechanical faults and prolonged downtimes in radiotherapy LINACs.

Determining suitable alternatives or updates to multileaf collimator designs requires a representative data sample of MLC operation in radiotherapy systems in LMICs, and these results provide an attempt to produce this dataset. Recommendations for a more comprehensive method for record keeping and potentially reducing the number of leaves have been motivated. Providing better cancer care must entail a reassessment of the MLC to account for discrepancies in LINAC downtimes and failure modes between LMICs and HICs.

## CONFLICT OF INTEREST

The authors declare no conflict of interest.

## AUTHOR CONTRIBUTIONS

*Guarantor of integrity of the entire study*: Suzie Sheehy. *Study concepts and design*: Gregory Peiris, Suzie Sheehy, Supriyanto Pawiro and Muhaammad Kasim. *Literature research*: Gregory Peiris. *Experimental studies/data analysis*: Gregory Peiris. *Statistical analysis*: Gregory Peiris. *Manuscript preparation*: Gregory Peiris and Suzie Sheehy. *Manuscript editing*: Gregory Peiris, Suzie Sheehy, Supriyanto Pawiro and Muhaammad Kasim.

## Data Availability

The data that support the findings of this study are available from the corresponding author upon reasonable request.

## Footnotes

1 This workshop was generously supported by the University of Oxford Global Challenges Research Fund (GCRF) and enabled by the Indonesian Ministry for Health.

## APPENDIX A

### A.1

Here, we detail the calculation of error bars based on uncategorised data points. The maximum possible contribution to the results from these uncategorised data points would be if they all belonged to the MLC, this would add a quantity ∆_MLC_ to the number of MLC faults and is used to calculate the upper bound error bar, as per below. The minimum possible contribution would be if all uncategorised faults were in the LINAC, but not the MLC subsystem, which would add ∆_LINAC_ faults to the total system (but zero to the MLC). The minimum and maximum error bars are thus calculated as:

$$\min=\frac{F_{\mathrm{MLC}}}{F_{\mathrm{LINAC}}+\Delta_{\mathrm{LINAC}}}\;\;\;\max=\frac{F_{\mathrm{MLC}}+\Delta_{\mathrm{MLC}}}{F_{\mathrm{LINAC}}},$$

where *F*_MLC_ is the number of MLC faults of a given subcategory and *F_LINAC_* are all faults of the LINAC. ∆_LINAC_ and ∆_MLC_ are the number of uncategorised faults of the whole dataset and MLC subset, respectively. This is why the error bars of *Cabling* in Figure 3 exceed the height of the bar.
